# Washing with hope: evidence of improved handwashing among children in South Africa from a pilot study of a novel soap technology

**DOI:** 10.1186/s12889-018-5573-8

**Published:** 2018-06-07

**Authors:** Justine Burns, Brendan Maughan-Brown, Âurea Mouzinho

**Affiliations:** 1 School of Economics, University of Cape Town, Private Bag, Rondebosch, 7701 South Africa; 2 Southern Africa Labour and Development Research Unit, University of Cape Town, Private Bag X3, Rondebosch, 7701 South Africa; 3 Abdul Latif Jameel Poverty Action Lab, University of Cape Town, Private Bag, Rondebosch, 7701 South Africa

**Keywords:** Handwashing intervention, Childhood health, Pilot randomised controlled trial, Behavioural economics, Development economics, Habit formation

## Abstract

**Background:**

While regular handwashing effectively reduces communicable disease incidence and related child mortality, instilling a habit of regular handwashing in young children continues to be a challenging task, especially in developing country contexts. This randomised controlled pilot study assessed the effect of a novel handwashing intervention – a bi-monthly delivery of a colourful, translucent bar of soap with a toy embedded in its centre (HOPE SOAP**©**) – on children’s handwashing behaviour and health outcomes.

**Methods:**

Between September and December 2014, 203 households in an impoverished community in Cape Town, South Africa, were randomised (1:1) to the control group or to receive HOPE SOAP**©**. Of all children (*N* = 287) aged 3–9 years and not enrolled in early childhood development programmes, 153 residing in intervention households received a bar of HOPE SOAP**©** every two weeks (total of 4 bars). Children in control households received a colourful, translucent bar of soap of equal size to HOPE SOAP**©**, with a toy alongside it. Two ‘snack tests’ (children were offered crackers and jam) were used to provide objective observational measures of handwashing. Through baseline and endline surveys, data were collected from caregivers on the frequency (scale of 1–10) of handwashing by children after using the toilet and before meals, and on soap-use during handwashing. Data on 14 illnesses/symptoms of illness experienced by children in the two weeks preceding the surveys were collected. Multivariable Ordinary Least Squares regression analyses were used to assess the intervention effect on handwashing behaviours and health outcomes.

**Results:**

At endline, HOPE SOAP**©** children were directly observed as being more likely to wash their hands unprompted at both snack tests (49% vs 39%, *β*: 0.10, *p* = 0.27). They were more likely to score ≥8/10 for using soap when washing their hands (*β*: 0.14, *p* = 0.011). HOPE SOAP**©** children, in general, had better health outcomes, and those who used the soap as intended, and did not cheat to remove the toy from the soap, were less likely to have been ill (*β*: − 0.15, *p* = 0.049).

**Conclusions:**

Results point towards HOPE SOAP© being an effective intervention to improve handwashing among children. Further research on this novel handwashing intervention is warranted.

**Trial registration:**

NCT03280771 (www.clinicaltrials.gov) retrospectively registered on 8 September 2017.

**Electronic supplementary material:**

The online version of this article (10.1186/s12889-018-5573-8) contains supplementary material, which is available to authorized users.

## Background

Preventable disease continues to be a leading cause of child deaths around the world. Diarrhoea and acute respiratory infections are responsible for 35 and 32% of child deaths respectively [1], whilst communicable diseases have been estimated to be responsible for 73% of deaths of children under the age of five years [2]. Furthermore, early childhood disease has been shown to have potential long-term human and economic costs by impacting future school readiness and achievement, outcomes in early adulthood, and performance on cognitive tests in old age [3–7].

Regular handwashing holds promise as a cheap and effective way to reduce communicable disease incidence and related child mortality in developing countries [8]. However, instilling a habit of regular handwashing in young children continues to be a challenging task, especially in developing country contexts, where it has been estimated in various studies that only 3–35% of individuals wash their hands at critical times [9]. The available literature suggests a number of key insights that are important in this regard. The first is that, on its own, the provision of information about the importance of handwashing may be necessary but not sufficient to lead to improved health outcomes. For example, mass media or information campaigns to promote handwashing appear to be largely ineffective [8, 10]. Recent work in behavioural science suggests that the failure of these interventions may stem from too great a reliance on conscious, deliberate thought and assimilation of knowledge to lead to behaviour change, when, in fact, many of these desired behaviours are automatic, largely unreflective and prompted by contextual cues [11–13]. Thus, if the goal is to induce habit formation, greater attention must be paid to System I thinking, which is far more automatic, unconscious and cue-driven [12]. For example, when soap and water are readily available, thereby providing a critical behavioural cue [12, 14], handwashing behaviour and health outcomes tend to improve on average [15, 16]. Inducing desired behaviour among children may further require leveraging context to produce a teachable moment [8], such as teaching proper handwashing technique and promoting the importance of handwashing at key times, namely prior to food preparation and after toilet use [12, 17].

The difficulty in translating interventions into behavioural change among children, and ultimately improved health outcomes among children, stems from the multiple factors that need to be aligned for success. For example, caregivers may fail to effectively transfer information from education campaigns to children. These difficulties in handwashing campaigns raise the possibility that targeting children directly may be more effective to achieve behavioural change. In addition, many handwashing studies rely on self-reported measures of handwashing behaviour which may be subject to bias. Obtaining objectively verified observational measures of handwashing is a challenge for any study of handwashing behaviour, with proposed solutions ranging from the use of costly acceleration sensors to direct observation. However, typical methods employed to measure handwashing via direct observation may lead to an overestimate of handwashing by as much as 20% [18].

In this study we assessed the effect on handwashing and health outcomes of a novel glycerine bar of soap, HOPE SOAP©, that included a child-friendly toy at the centre which could be accessed through regular handwashing. We tested a novel data collection technique designed to minimize bias in typical handwashing measures by adding a ‘snack test’ to a pre-existing programme, and using the programme implementers already familiar to children to collect a directly observable measure of handwashing. Thus, by relying on a randomised controlled trial in conjunction with a minimally-intrusive, directly observable measure of handwashing behaviour, this study hopes to overcome some of the difficulties that have plagued previous studies. In addition, this study adds to the limited experimental evidence in an African context on the efficacy of a handwashing intervention targeting children directly.

## Methods

Our study was conducted between September and December of 2014 in the impoverished community of Delft in the Western Cape, South Africa. Delft is a township on the outskirts of Cape Town (approximately 34 km from the centre of the city) consisting largely of government housing projects within an area of approximately 11 km^2^. The study was implemented in partnership with the Foundation for Community Work (FCW), an early childhood development and resource organisation. FCW runs an in-home education programme called Family-in-Focus, which involves fortnightly home-visits conducted by trained community workers, during which they engage with caregivers and their children, sharing knowledge on child development and facilitating activities to promote caregiver-child interaction. The community of Delft was selected as the pilot site because it has been identified by the provincial Department of Health with a high burden of diarrhoea [19], and due to the large number of households and children in the area enrolled in the Family-in-Focus programme. At the start of the implementation period, the study site was served by 13 community workers.

Eligibility for inclusion in the pilot was based on the following criteria: (1) the caregiver was participating in the FCW Family-in-Focus programme; (2) the caregiver had at least one child between the ages of three and nine years old in the programme; (3) the age-eligible children were not involved in any other sort of early childhood development programme (eg, crèche or other day-care). Eligibility was based on caregivers and not households, since a single household could contain more than one caregiver. The child age-bracket was based on the fact that FCW do not work with children older than nine, and that they only start targeting children with hygiene and handwashing messages once they turn three.

### The intervention: HOPE SOAP©

HOPE SOAP©^1^ is a colourful, translucent bar of soap with a toy embedded in its centre. The aim of this innovative yet simple soap technology is to encourage handwashing practice among children by making it fun and goal-oriented. HOPE SOAP© is smaller than typical bars of soap used by households in the study area, with dimensions of 50 millimetres (mm) by 44 mm by 24 mm. A range of toys, such as a bouncing ball and a plastic fish, were used.

### The control group soap

Children in the control group received soap with the same specifications as HOPE SOAP© (a colourful, translucent bar of the same size), but there was no toy embedded in the centre of the control group soap. Since households assigned to treatment and control arms of the study could live close to one another, this raised the possibility that children in control households would learn about HOPE SOAP© and possibly become disgruntled by not receiving a toy and thus be less inclined to use their soap, or conversely, might try to assist children in treatment households in getting the toy out of the soap by also using it. To minimise these behaviours, and associated potential confounds on our treatment effect, children in control households received the control group soap together with a toy alongside the soap. These toys were the same as those placed inside HOPE SOAP©. Thus, the control group soap was also smaller than typical soap used in the area, and came in a greater variety of colours. We refer to this soap as ‘control’ soap.

### Randomisation

Using a randomised trial, our pilot study was designed to test whether HOPE SOAP©, in comparison to ‘control’ soap, increases handwashing among young children, both in the short-term and in the long-term (ie, habit formation), and results in better health outcomes. Because assignment to treatment was random, receipt of the intervention was exogenous, and therefore unrelated to any other observable and unobservable factors [20]. This solves problems of selection bias and unobserved heterogeneity. The sample frame for this study consisted of all residential addresses in Delft (*N* = 203) provided by FCW at which a child (3–9 years old) was enrolled in their Family-in-Focus programme. All caregivers enrolled in the Family-in-Focus programme at these residences participated in our study. Since intra-household spillovers in soap usage would be difficult to prevent and measure, we randomised (1:1) to either HOPE SOAP© or the control soap at the household level (*n* = 203) using the statistical software package Stata 14 (Stata Corporation LP, College Station, TX). All study eligible children residing in each household were assigned to the same study group. We stratified the randomisation by community worker, household size, the gender and age ratio among eligible children, and the number of caregivers in the household. Although a third group of comparison households in which eligible children received nothing would have been ideal, binding budget constraints for the pilot made this impossible.

### Intervention delivery

**Fig. 1:**
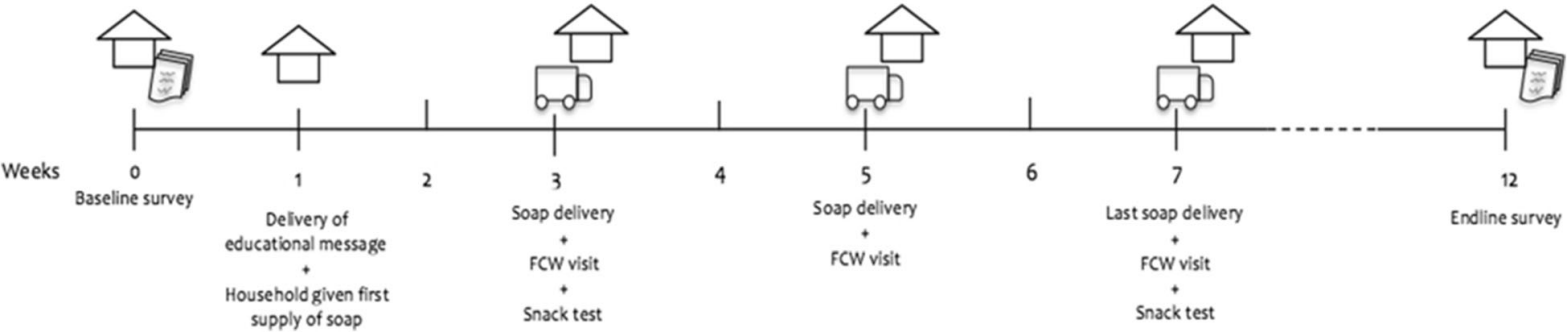
Timeline of intervention and data collection for the HOPE SOAP**©** pilot study

The Family-in-Focus programme formally addresses issues of child health and handwashing in its content, providing an ideal platform to roll out the HOPE SOAP© pilot intervention. Figure 1 outlines the schedule of soap deliveries. The first delivery of soap into households was made by community workers, one week after the baseline survey, to the families assigned to them in the Family-in-Focus programme. Before giving the first bar of soap to children, the community workers provided the single standard lesson on health and hygiene that was designed for the Family-in-Focus programme (see Additional file 1 for details on the content of the lesson). The single health and hygiene lesson was provided to all caregivers and children (ie, in both the treatment and control households) to ensure equal exposure to this information at the start of the intervention. The soap was packaged in brown paper parcels by the research team and labelled with the child’s name to ensure (1) that a sense of ownership was created for children, and (2) that community workers were blinded to group assignment while providing the education lesson.

Subsequent to the first delivery of soap, the intervention consisted of fortnightly provision of HOPE SOAP© bars to children in treatment households and control soap with a toy alongside to children in control households over a 6-week period. All children in the study received four bars of soap. All soap was labelled with the child’s name and packaged in brown paper parcels by the research team. These soap deliveries were conducted by an independent team (ie, not the community workers) so as to ensure that study group assignment remained fixed throughout the study. Deliveries were typically made at a time when community workers were not visiting families. Soap was delivered to children’s caregivers and no additional health education was provided.

### Data and measures

Figure 1 provides the timeline for data collection. A baseline survey was conducted in September 2014 to collect household characteristics (ie, composition, infrastructures, assets, health), knowledge about health and hygiene practices, as well as attitudes and behaviour towards handwashing. Additional observational data on soap availability and household cleanliness were also collected. An endline survey was conducted shortly after the intervention period ended in December 2014. The baseline survey was repeated with additional data being collected on usage of and attitude towards HOPE SOAP©. The baseline and endline surveys were administered by an independent research team (ie, not by community workers).

The baseline and endline surveys collected self-reported data from caregivers on the frequency of soap use and handwashing by children at two critical times, namely, after the toilet and before meals. Specifically, caregivers were asked ‘On a scale of 1 to 10, where 1 means never and 10 means always, how regularly does […] wash his or her hands without being prompted after using the toilet?’ and ‘On a scale of 1 to 10, where 1 means never and 10 means always, how regularly does […] wash his or her hands without being prompted before a main meal?’ Caregivers used the same scale to report how often the child used soap when he or she washes his or her hands. Data on child health outcomes in the two weeks preceding the survey were also collected by asking caregivers whether each child had experienced any of the following in the two weeks prior to each survey: blocked nose, runny nose, persistent sneezing, sore or scratchy throat, painful swallowing, cough, fever, headache, shortness of breath, itchy and watery eyes, nausea, vomiting, rash, diarrhoea. An illness score (0–14) was created as the sum of the number of symptoms experienced.

Between the baseline and endline surveys the community workers conducted a ‘snack test’ during two of the four scheduled home-visits. Here, the community worker offered a snack of crackers and jam to the children at the end of the home-visit and observed whether or not the children washed their hands unprompted before eating the snack. We use this data as our objective observational measure of handwashing to complement the caregiver-reported measures of handwashing collected during the surveys. As it was impossible to completely blind community workers to the study group assignment – community workers would return to households as part of the regular Family-in-Focus programme and could observe which soap was in a household – we attempted to minimise potential treatment effect bias associated with the non-blinded study in three ways. First, we stratified our randomisation by community workers to ensure that community workers had children in both treatment and control households. Measurement bias resulting from community workers recording a more positive outcome in order to show that her households were doing well would therefore apply for both treatment and control households, and not influence findings on the treatment effect. Second, community workers were under the impression that the study aimed to assess the impact of soap provision to households, rather than the impact of HOPE SOAP© relative to normal soap specifically. Third, all deliveries of soap were made in brown parcels, and deliveries of soap subsequent to the first delivery were made by an independent research team at a time when the community worker was not with households. This step aimed to keep group assignment as obscure as possible to the community workers, and minimise the salience of the study in their minds.

### Analysis

We first present baseline sample characteristics by assignment to study group to assess whether there were any differences in key characteristics following randomisation. Ordinary least squares regression models were used to estimate the effect of assignment to HOPE SOAP© on observed handwashing during the snack tests, as well as endline measures of 1) frequency of handwashing after the toilet and before meals; 2) frequency of soap usage when washing hands; 3) and number of symptoms of illness experienced. All standard errors were clustered by FCW community worker. By accounting for clustering at this level, we control for all potential error correlation between families served by the same FCW community worker. This includes potential correlation between family units living within the same physical structure. To increase the precision of the estimates in the multivariable models, we controlled for key demographics, and factors that could influence handwashing that had a relatively large difference (*p* < 0.2) across study groups at baseline.

## Results

### Baseline characteristics

Our baseline sample was recruited from 203 houses (ie, physical structures). In 22 cases, multiple caregivers with children enrolled in the FCW programme resided in the same house. These caregivers defined their family unit as a separate household despite living in the same physical structure. Accordingly, for our analysis, we defined households as separate family units rather than physical structures. Tables 1 and 2 presents baseline characteristics of the households (*N* = 229), caregivers (*N* = 229) and children (*N* = 288) in our sample.

**Table 1:** Baseline summary statistics: households and caregivers

	Full sample			Treatment		Control		Treatment-Control	
	n	Mean	SD	n	Mean	n	Mean	Difference	*p*-value
Household Characteristics									
Number of members	229	6.47	2.64	123	6.42	106	6.52	−0.10	0.799
Number of children (2 to 10 yrs. old)	229	2.27	1.22	123	2.19	106	2.36	−0.17	0.324
Race of household head: Coloured^a^	219	0.82	0.38	120	0.83	99	0.82	0.01	0.900
Household head: completed grade 12	189	0.14	0.35	105	0.13	84	0.14	−0.01	0.855
Any member receives a govt. grant	229	0.89	0.31	123	0.92	106	0.86	0.06	0.151
Asset index (0–20)	223	9.16	3.15	121	9.13	102	9.19	−0.05	0.906
Monthly household income <R2000	214	0.41	0.49	113	0.42	101	0.40	0.02	0.779
Piped water in house	228	0.79	0.41	123	0.73	105	0.85	−0.12	0.041
Toilet in house	226	0.87	0.34	122	0.84	104	0.90	−0.06	0.196
Household limits water use^b^	214	0.44	0.50	110	0.50	104	0.39	0.12	0.096
Hygiene & health									
Household uses bar soap	226	0.93	0.26	121	0.93	105	0.92	0.00	0.962
Soap always available (self-report)	226	0.72	0.45	121	0.73	105	0.71	0.02	0.716
Soap observed in house by fieldworker	198	0.61	0.49	104	0.56	94	0.66	−0.10	0.156
Household limits handwashing^c^	228	0.18	0.38	123	0.17	105	0.19	−0.02	0.701
Any household member ill in past 2 weeks	229	0.65	0.48	123	0.62	106	0.68	−0.06	0.354
Any child ill in past 2 weeks	229	0.42	0.49	123	0.38	106	0.46	−0.08	0.248
Caregiver characteristics									
FIF Programme participant > 3 months^d^	222	0.62	0.49	120	0.58	102	0.67	−0.08	0.215
Received hygiene training in past 3 months	220	0.61	0.49	119	0.56	101	0.65	−0.09	0.183
Reported handwashing prevents diarrhoea^e^	229	0.61	0.49	123	0.60	106	0.61	−0.01	0.862
Handwashing technique knowledge (0–40)^f^	226	32.40	7.33	121	32.11	105	32.73	−0.63	0.517
Washes hands before cooking & eating	229	0.78	0.42	123	0.79	106	0.76	0.02	0.659
Always uses soap to wash hands	228	0.80	0.40	123	0.80	105	0.80	0.00	0.950
Health never affects activities	228	0.57	0.50	122	0.53	106	0.60	−0.07	0.278
Depressed/anxious 3+ days in past week	229	0.56	0.50	123	0.60	106	0.51	0.09	0.155

*SD* Standard deviation

^a^‘Coloured’ is a commonly used racial classification in South Africa, which describes an individual of mixed-race ancestry

^b^Caregivers were asked ‘Can this household afford to use as much water as it needs every month, or do you have to limit your usage in any way?’

^c^Caregivers were asked whether their household limits handwashing to limit the amount of water used

^d^This indicator represents caregivers who had been participating in the Family-in-Focus (FIF) programme for more than three months

^e^Caregivers were asked the open-ended question: ‘How do you think diarrhoea can be prevented?’

^f^Using a scale of 1 to 10 (1: not important, 10: very important) caregivers were asked the importance of four activities during handwashing: using soap, using warm water, rubbing hands together, lathering soap. The four responses were summed

**Table 2:** Baseline summary statistics: children

	Full sample			Treatment		Control		Treatment-Control	
	n	Mean	SD	n	Mean	n	Mean	Difference	*p*-value
Female	287	0.49	0.50	153	0.49	134	0.49	0.00	0.970
Age	287	4.48	1.36	153	4.46	134	4.51	− 0.04	0.798
*Handwashing barriers* ^a^									
Too short to reach tap	284	0.35	0.48	151	0.39	132	0.30	0.10	0.152
Can’t open tap	284	0.38	0.49	150	0.45	133	0.30	0.15	0.026
Hands too small for soap	284	0.32	0.47	150	0.37	133	0.26	0.11	0.077
Water too hot/cold	287	0.19	0.39	153	0.20	134	0.16	0.04	0.502
Dirty water	287	0.18	0.38	153	0.18	134	0.16	0.02	0.739
Water smells bad	287	0.16	0.37	153	0.16	134	0.16	0.00	0.998
Household water saving	284	0.15	0.36	150	0.14	133	0.16	−0.02	0.751
Sounds from water tap	284	0.06	0.24	150	0.09	133	0.04	0.05	0.204
*Handwashing*									
After toilet (1–10)	282	5.73	3.00	151	5.62	130	5.89	−0.28	0.501
Before meals (1–10)	283	5.35	2.95	150	5.16	132	5.60	−0.44	0.299
Uses soap (1–10)	275	6.93	2.79	144	6.95	130	6.89	0.07	0.870
*Health*									
Illness score (0–14)	249	2.55	2.59	137	2.51	112	2.59	−0.08	0.835
Any illness symptom	249	0.74	0.44	137	0.77	112	0.71	0.07	0.270
2+ illness symptoms	249	0.57	0.50	137	0.55	112	0.59	−0.04	0.580

*SD* Standard deviation

^a^Caregivers reported how often different factors affected their child’s willingness to wash his or her hands. A binary variable was created for each factor with 1 = all, most, or some of the time; and 0 = none of time

Of the 229 households/family units that were surveyed at baseline, 123 households were randomly assigned to treatment. The vast majority of household heads had not completed high school (86%), and there was high reliance on government social welfare grants. Despite generally low monthly household income, basic hygiene-related infrastructure was good: 79% of households had piped water in the house and 87% reported a flush toilet within the dwelling. In terms of household hygiene (Table 1), almost all households were using bar soap. The provision of bar soap to households during our study would therefore not have introduced an unfamiliar soap product. Just over a quarter of households (28%) reported not having soap always available and in more than a third (39%) of households, the fieldworker did not observe soap for handwashing on the day of the baseline survey. Furthermore, just under a fifth of households reported that they limited handwashing activities in some way due to water scarcity. Reports of illness within the household were common with 65% of households reporting that at least one household member had diarrhoea, flu or nausea in the 2 weeks preceding the baseline survey, and 42% reporting the same for children.

The caregivers (Table 1) of the children in our sample had, on average, been involved with the FCW programme for a relatively short time with a mean of 5 months and 38% having enrolled in the three months prior to the baseline. Self-reported knowledge of how to effectively wash hands was high among caregivers, with an average score of 32 out of a maximum of 40 based on knowledge of four components of handwashing (the importance of soap, warm water, rubbing hands and lathering). However, 39% did not mention handwashing as a means to prevent diarrhoea, over a fifth reported that they did not always wash hands before cooking and eating (22%), and 20% did not always use soap when washing hands.

Our sample of children (Table 2) comprised similar numbers of girls and boys, with an average age of four and a half. For approximately a third of children, being too short to reach the tap, having difficulty opening the tap and having hands too small to hold the soap were reported to affect handwashing most or all of the time. Factors relating to the water supply (temperature, cleanliness and smell) were also potential barriers to handwashing. In terms of handwashing behaviour, unprompted handwashing was relatively infrequent at baseline with an average of less than six on the handwashing scale (1–10:1 = never, 10 = always) for both scenarios of handwashing after the toilet and before meals. In cases when children did wash their hands, the average score for the regularity of soap use during handwashing was 6.9, with 49% of the children scoring an 8 or above.

A key feature to note in Tables 1 and 2 is that in almost all cases, sample attributes were balanced between the treatment and control group, with exceptions being that treatment households were significantly less likely to have piped water in the household, and were more likely to limit water use. In addition, children in treatment households were significantly more likely to be reported as having difficulty opening a tap, and as having hands that were too small to hold regular soap.

### HOPE SOAP**©** children were directly observed as being more likely to wash their hands unprompted prior to eating a snack, although the differences were not statistically significant

**Fig. 2:**
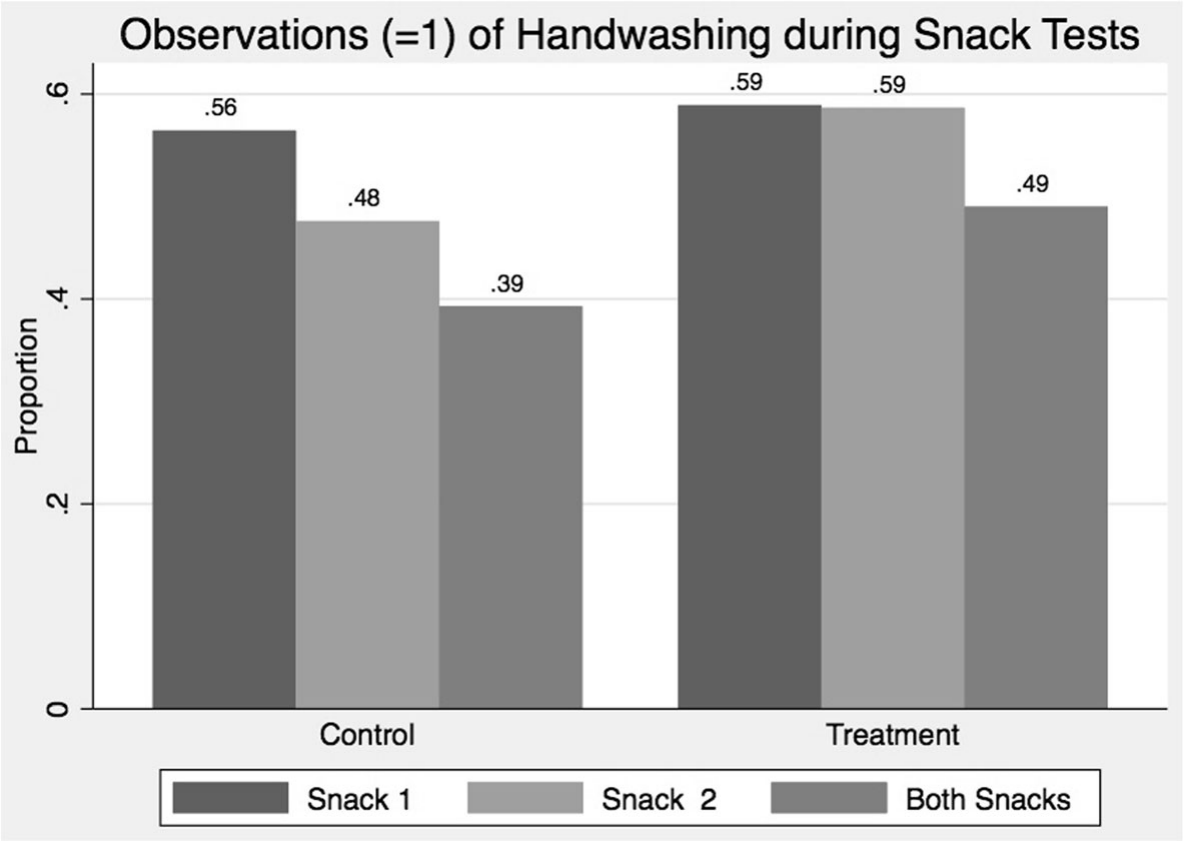
Observations of handwashing among children during the snack tests

Figure 2 displays the proportion of children in treatment and control groups respectively who, without being prompted, washed their hands before eating at the first and second snack test. Similar proportions of children in both groups were observed to wash their hands prior to the first snack test (control: 56%, treatment: 59%). However, differences were evident by the second snack test. At snack test two, 59% of children in the HOPE SOAP**©** group washed their hands compared to 48% in the control group (*p* = 0.215), indicating an 8% point decrease in handwashing at snack test two among children in the control group.

After controlling for demographic characteristics and factors that were unbalanced between treatment and control groups at baseline, treatment children were 6% points (*p* = 0.485) more likely to wash their hands before eating at snack test two (Table 3, Model 4). Moreover, children in the HOPE SOAP**©** group were 10% points (*p* = 0.264) more likely to wash their hands before both snack tests compared to control group children (Table 3, Model 6). Sensitivity analysis using multivariable logistic regression models with the same specifications as the models presented in Table 3 found substantively similar results. The adjusted odds ratio for the treatment effect on washing hands at both snack tests (equivalent to Model 6, Table 3) was 1.53 (*p* = 0.279).

**Table 3:** Ordinary least squares regression models of treatment effects on observed handwashing (yes = 1, no = 0) among children during the snack tests

	1	2	3	4	5	6
	Washed at Snack 1	Washed at Snack 1	Washed at Snack 2	Washed at Snack 2	Washed at Snack 1&2	Washed at Snack 1&2
Treatment: HOPE SOAP**©**	0.02^**a**^	0.04	0.11	0.06	0.10	0.10
	(−0.13–0.18)	(−0.12–0.20)	(−0.06–0.29)	(−0.11–0.23)	(−0.08–0.27)	(−0.07–0.27)
Controls	No	Yes	No	Yes	No	Yes
Observations	230	228	188	187	188	187
R-squared	0.00	0.12	0.01	0.17	0.01	0.13

^a^Beta coefficient presented followed by 95% confidence intervals in parentheses

*** *p* < 0.01, ** *p* < 0.05, * *p* < 0.1

Additional controls included but not reported: female, age, household size, number of children in households (HH), asset ownership, piped water available in HH, HH limits water use; soap observed in HH; HH received hygiene training; caregiver depressed/anxious; child had difficulty opening tap; child cannot reach taps; child’s hands too small for soap. The full model, with coefficients for all control variables, is presented in Additional file 1: Table S1

### Reported handwashing improved in both study groups

**Fig. 3:**
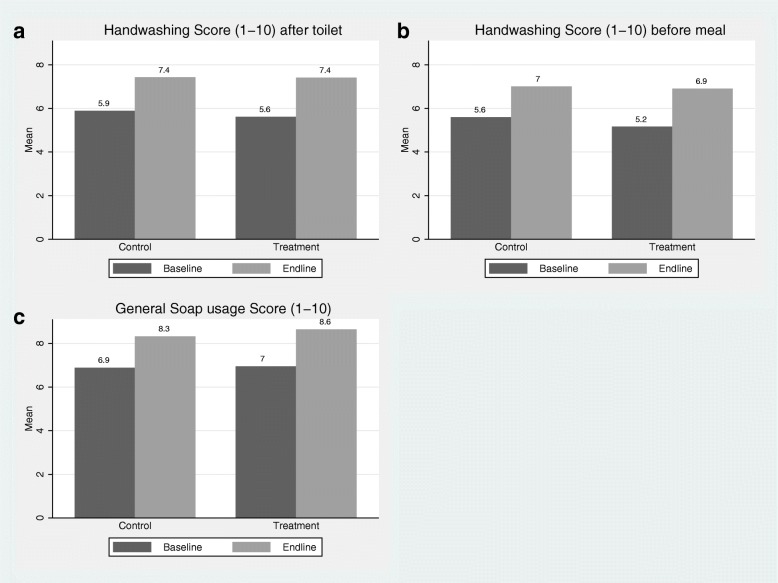
Child handwashing behaviour as reported by caregivers: **a** handwashing after using the toilet; **b** handwashing before meals; **c** soap-use during handwashing

Figure 3 displays the change between baseline and endline in the average score for three different caregiver-reported handwashing measures. Overall, in both groups, substantial improvements in handwashing were found for both the frequency of handwashing at critical times and the use of soap when hands were washed. At endline, according to bivariate analyses, there were no significant differences in these measures among control and treatment children.

### Conditional on poor baseline handwashing behaviour, HOPE SOAP**©** children were more likely to wash their hands after using the toilet

The results for the effect of HOPE SOAP**©** on handwashing after going to the toilet are presented in Table 4, Panel A. A weak positive treatment effect was found after controlling for baseline factors (Models A2 & A3). However, Model A4 indicates that the treatment effect varied according to baseline handwashing score with a larger effect among children who were initially relatively poorer handwashers (treatment coefficient: 1.29, *p* = 0.117), and a weakening treatment effect among initially better handwashers (interaction term: − 0.19, *p* = 0.093). Model A5 shows a positive, but not statistically significant association between receipt of HOPE SOAP**©** and the proportion at the top end of the handwashing score. Similar results are found in relation to the effect of HOPE SOAP**©** on handwashing before meals as reported in Table 4, Panel B, although the results lack statistical power.

**Table 4:** Ordinary least squares regression models of treatment effects on handwashing outcomes among children (as reported by caregivers)

Panel A: Handwashing after toilet	A1	A2	A3	A4	A5
	Handwashing score [1–10]	Handwashing score [1–10]	Handwashing score [1–10]	Handwashing score [1–10]	≥8 on scale [0/1]
Treatment: HOPE SOAP**©**	− 0.01^a^	0.11	0.20	1.29	0.08
	(−0.69–0.67)	(−0.62–0.84)	(−0.49–0.88)	(−0.33–2.91)	(−0.06–0.23)
^b^Baseline handwashing measure			0.22***	0.32***	0.29***
			(0.10–0.34)	(0.15–0.50)	(0.15–0.43)
Treatment*Baseline handwashing measure				−0.19*	
				(−0.41–0.03)	
Controls	No	Yes	Yes	Yes	Yes
Observations	249	247	242	242	242
R-squared	0.00	0.08	0.16	0.17	0.16
Panel B: Handwashing before meal	B1	B2	B3	B4	B5
	Score 1–10	Score 1–10	Score 1–10	Score 1–10	≥8 on scale
Treatment: HOPE SOAP**©**	−0.10	0.27	0.28	1.14	0.05
	(−0.86–0.65)	(− 0.49–1.03)	(−0.46–1.02)	(− 0.46–2.73)	(− 0.08–0.19)
^b^Baseline handwashing measure			0.13**	0.22**	0.10
			(0.01–0.25)	(0.04–0.39)	(− 0.05–0.26)
Treatment*Baseline handwashing measure				− 0.16	
				(−0.40–0.09)	
Controls	No	Yes	Yes	Yes	Yes
Observations	249	247	244	244	244
R-squared	0.00	0.09	0.11	0.11	0.12
Panel C: General soap usage when handwashing	C1	C2	C3	C4	C5
	Score 1–10	Score 1–10	Score 1–10	Score 1–10	≥8 on scale
Treatment: HOPE SOAP**©**	0.31	0.42*	0.41	0.91	0.14**
	(−0.27–0.89)	(−0.07–0.91)	(− 0.09–0.90)	(− 0.89–2.70)	(0.03–0.24)
^b^Baseline handwashing measure			0.13**	0.17*	0.15**
			(0.01–0.26)	(−0.02–0.36)	(0.02–0.28)
Treatment*Baseline handwashing measure				− 0.07	
				(−0.31–0.17)	
Controls	No	Yes	Yes	Yes	Yes
Observations	249	247	236	236	236
R-squared	0.01	0.07	0.10	0.10	0.13

^a^Beta coefficient presented followed by 95% confidence intervals in parentheses

^b^Baseline equivalent of dependent variable

*** *p* < 0.01, ** *p* < 0.05, * *p* < 0.1

Additional controls included but not reported: Female, age, household size, number of children in HH, asset ownership, piped water available in HH, HH limits water use; soap observed in HH; HH received hygiene training; caregiver depressed/anxious; child had difficulty opening tap; child cannot reach taps; child’s hands too small for soap. The full models, with coefficients for all control variables, are presented in Additional file 1: Tables S2, S3 and S4

### HOPE SOAP**©** children were significantly more likely to use soap when washing their hands

A more positive treatment effect was found on general soap use when children did wash their hands (Table 4, Panel C). The average soap use score was 0.42 higher (*p* = 0.094) among children in the HOPE SOAP**©** group than control children after including control variables (Model C2). At the top end of the scale (Model C5), the proportion scoring higher than eight was 14% points greater (*p* = 0.011) in the treatment group. Sensitivity analysis using a multivariable logistic regression model with the same specification as Model C5 also showed that children in the treatment group were significantly more likely to use soap when washing hands (adjusted odds ratio: 2.42, *p* = 0.017).

### HOPE SOAP**©** children exhibited better health outcomes on average, although the differences were not statistically significant

A reduction in symptoms of illness experienced was observed for both treatment and control children. The average of the illness score (0–14) decreased from 2.5 to 1.7 among HOPE SOAP**©** children and from 2.6 to 1.9 among children in the control group, with the average illness score marginally lower among treatment children at endline. A small treatment effect on health remained after controlling for baseline characteristics (Table 5). The average illness score was − 0.35 lower among HOPE SOAP**©** children (Model 3, *p* = 0.256) and the proportion of HOPE SOAP**©** children who experienced any symptom of illness in the two weeks prior to the endline survey was 9% points lower (Model 7, *p* = 0.204).

**Table 5:** Ordinary least squares regression models of treatment effects on experience of illness

	1	2	3	4	5	6	7
	Illness score [1–14]	Illness score [1–14]	Illness score [1–14]	Illness score [1–14]	Any illness [0/1]	Any illness [0/1]	Any illness [0/1]
Treatment: HOPE SOAP**©**	−0.20^a^	− 0.28	−0.35	− 0.10	−0.06	− 0.06	−0.09
	(−0.79–0.39)	(−0.86–0.31)	(−0.96–0.26)	(−0.82–0.62)	(−0.19–0.08)	(−0.18–0.07)	(−0.22–0.05)
Baseline illness measure			0.19**	0.24**			0.16**
			(0.04–0.34)	(0.02–0.47)			(0.01–0.31)
Treatment*Baseline illness measure				−0.10			
				(−0.39–0.18)			
Controls	No	Yes	Yes	Yes	No	Yes	Yes
Observations	249	247	236	236	236	236	236
R-squared	0.01	0.07	0.10	0.10	0.09	0.07	0.13

^a^Beta coefficient presented followed by 95% confidence intervals in parentheses

*** *p* < 0.01, ** *p* < 0.05, *p < 0.1

Additional controls included but not reported: Female, age, household size, number of children in HH, asset ownership, piped water available in HH, HH limits water use; soap observed in HH; HH received hygiene training; caregiver depressed/anxious; child had difficulty opening tap; child cannot reach taps; child’s hands too small for soap. The full model, with coefficients for all control variables, is presented in Additional file 1: Table S5

### HOPE SOAP**©** children who used the soap as intended exhibited significantly better health outcomes compared to the control group

The intention of HOPE SOAP**©** was to encourage children to wash their hands more frequently in the process of retrieving the toy. However, children may have used other strategies to obtain the toy such as dissolving the soap in water or cutting the soap. In such cases children who received HOPE SOAP**©** could end up worse off than control children because of decreased access to soap. We create an indictor (‘toy-cheat’) using responses from caregivers about whether the child ever retrieved the toy by (1) dissolving the soap in water, (2) cutting the bar of soap, or (3) destroying the soap in another manner. Notably, this is a blunt indicator as caregivers would have had to both observe and report this behaviour, and they could have reported this behaviour even if it only happened on one occasion. According to this measure, 42% of the treatment children were classified as toy-cheats. There was no evidence that toy-cheats had less access to soap than other children who received HOPE SOAP©. Caregivers of toy-cheats and non toy-cheats reported a similar average for the number of days that a bar of soap lasted (8.4 vs 8.3).

Multiple regression models (Table 6) found that children who used the soap as intended (non toy-cheat) had a significantly lower illness score (Model 1) and were 15% points less likely (*p* = 0.049) to experience any illness in the two weeks prior to the endline survey compared to the control group (Model 2). In contrast, the difference in health between control group children and the toy-cheats was negligible. Sensitivity analysis using multivariable logistic regression analysis with the same specification as Model 2 also found that children in the treatment group who used HOPE SOAP© as intended were significantly less likely to have experienced any illness in the two weeks prior to the endline survey compared to the control group (adjusted odds ratio: 0.44, *p* = 0.039).

**Table 6:** Ordinary least squares regression models of treatment effects on handwashing and health by correct use of HOPE SOAP**©**

	(1)	(2)
	Illness score [1–14]	Any illness [0/1]
HOPE SOAP**©** (vs control)		
Not a Toy-Cheat	−0.61*	− 0.15**
	(− 1.31–0.09)	(−0.31 - -0.00)
Toy-Cheat	0.07	0.02
	(− 0.65–0.79)	(− 0.14–0.18)
Baseline illness measure	0.19**	0.16**
	(0.04–0.34)	(0.01–0.31)
Controls	Yes	Yes
Observations	209	209
R-squared	0.23	0.23

^a^Beta coefficient presented followed by 95% confidence intervals in parentheses

*** *p* < 0.01, ** *p* < 0.05, * *p* < 0.1

Additional controls included but not reported: female, age, household size, number of children in HH, asset ownership, piped water available in HH, HH limits water use; soap observed in HH; HH received hygiene training; caregiver depressed/anxious; child had difficulty opening tap; child cannot reach taps; child’s hands too small for soap. The full model, with coefficients for all control variables, is presented in Additional file 1: Table S6

## Discussion

There are a number of positive results from the HOPE SOAP**©** pilot study which suggest real potential for this innovative soap product to increase and sustain handwashing among young children. Conditional on poor baseline handwashing behaviour, HOPE SOAP**©** children were more likely to wash their hands after using the toilet, albeit insignificantly so, and were also significantly more likely to use soap to wash their hands as opposed to just rinsing with water. Furthermore, a greater proportion of children in the HOPE SOAP**©** group (10% points) were observed to wash their hands unprompted both times they were offered a snack. This suggests there is merit in making handwashing a fun and goal-oriented activity, especially for children who do not regularly wash their hands. Moreover, HOPE SOAP**©** children, in general, had better health outcomes and those who used the soap as intended, and did not cheat to remove the toy from the soap, evidenced significantly better health outcomes.

Whilst there are a number of other positive results on reported handwashing behaviour, our results lack statistical power. This could be due to a number of factors, the most obvious being small sample size which is an inevitable feature of most pilot studies. Attrition between baseline and endline further reduced the sample, although the attrition rate for households from our baseline sample was only 13,5% and differences between the control and treatment group were not significant.

Spillover effects, both between and within households, may also serve to dampen estimated treatment effects. Whilst we stratify on the basis of household size and the adult to child ratio within the household when conducting the randomisation, it still remains the case that if the novelty of HOPE SOAP**©** induced other household members within the household to wash their hands more regularly than household members in the control group, the soap may have been depleted more quickly, thereby undermining potential health benefits for treated children. This could contribute to reduced treatment effects. In contrast, we do not think that between household spillovers constitute a serious problem in this study as fewer than 2% of treated children were reported to have shared their soap with children outside the household.

The magnitude of our treatment effect is also undermined by substantial increases in handwashing among children in control households during the study period. Control group children received child-sized, bright, translucent bars of soap. This soap was different than the usual household soap, and may itself have induced children in control households to wash their hands more frequently. Our data shows that the proportion of children whose caregivers reported “small hands” as a barrier to handwashing halved between baseline and endline (32 to 15%). This effect was similar in both treatment and control groups suggesting that delivery of child-sized soap may itself be an important (and even sufficient) intervention to induce handwashing among children. Had control group children received normal household soap (typically larger in size and not as colourful), one might have anticipated larger treatment effects. Whilst we acknowledge that access to basic provision might sometimes act as a barrier to handwashing, in our sample, 80% of households had access to running water and flush toilets, making it difficult to argue that access to services was the key barrier to handwashing. Rather, it appears that lack of access to ‘child-friendly’ soap may constitute a more significant barrier in this particular instance. Further research is warranted as these findings point towards child-friendly soap, sized appropriately for ease of use by small hands, as a potential low-cost intervention to improve handwashing among children.

The overall health impacts associated with HOPE SOAP**©** were not particularly strong. Children in both treatment and control groups appear to have enjoyed improved health between baseline and endline. This could be due to the increased handwashing induced by the availability of soap, which our data certainly suggests, but it could also reflect seasonal changes that may be associated with improved health, since the baseline was conducted in Spring and the endline was conducted during the summer months. However, despite this confound, the fact that HOPE SOAP**©** children who used the soap as intended (ie, they did not cut or dissolve the soap to get the toy) enjoyed significantly better health outcomes compared to the control group is certainly encouraging. Furthermore, the health outcomes of children who were categorised as toy-cheats were similar to those in the control group, indicating that there were no unintended negative effects of the intervention on health.

Study results should be interpreted in the context of other potential limitations. It is possible that social desirability bias, recall bias and incorrect knowledge might have resulted in misreporting of handwashing and child health by caregivers during the baseline and endline surveys. However, there is no a priori reason to expect that this bias would be different across treatment and control groups. Although several measures were taken to minimise any bias resulting from community workers being non-blinded to study group assignment, it is possible that this introduced some bias in the estimation of the effect of HOPE SOAP**©**. This is a problem for any study that aims to collect observational measures of soap usage, our study is not unique, and we think such bias is likely to be minimal given our study design. In addition, the period between endline measurements and initiation of the intervention was relatively short. Further research is required to assess the impact of HOPE SOAP**©** on long-term habit formation.

## Conclusions

Overall, results indicate that HOPE SOAP**©** does, indeed, give cause for hope. There is evidence that it may improve, and induce sustained handwashing behaviour among young children and, if used correctly, improve health outcomes. If HOPE SOAP**©** can be produced and sold at the same price as regular soap it could well prove to be a more effective intervention than simple soap distribution. The results of our pilot study indicate that further research on this novel handwashing intervention is warranted.

## Additional file

Additional file 1: Hygiene education message. **Table S1.** Ordinary least squares regression models of treatment effects on handwashing during the Snack Tests. **Table S2.** Effect of treatment on handwashing after going to the toilet. **Table S3.** Effect of treatment on handwashing before meals. **Table S4.** Effect of treatment on general soap usage when washing hands. **Table S5.** Effect of treatment on child health. **Table S6.** Effect of treatment on handwashing and health by correct use of HOPE SOAP**©. (DOCX 75 kb)**

## Abbreviation

FCW: Foundation for Community Work

## Footnotes

1 HOPE SOAP© was created through a partnership between advertising agency Young & Rubicam (Y&R) and Safety Lab, a non-profit behavioural innovation lab.
