# Supplementary material for: Washing with hope: evidence of improved handwashing among children in South Africa from a pilot study of a novel soap technology
Source: BMC Public Health. 2018 Jun 7;18:709. doi: 10.1186/s12889-018-5573-8 (PMC5992781; doi:10.1186/s12889-018-5573-8)
Supplement: Supplementary file 1 — Hygiene education message. Table S1. Ordinary least squares regression models of treatment effects on handwashing during the Snack Tests. Table S2. Effect of treatment on handwashing after going to the toilet. Table S3. Effect of treatment on handwashing before meals. Table S4. Effect of treatment on general soap usage when washing hands. Table S5. Effect of treatment on child health. Table S6. Effect of treatment on handwashing and health by correct use of HOPE SOAP©. (DOCX 75 kb) [file 12889_2018_5573_MOESM1_ESM.docx]

**Additional file 1**

**Article “**Washing with Hope: Evidence of improved handwashing among children in South Africa from a pilot study of a novel soap technology”

Justine Burns, Brendan Maughan-Brown, and Âurea Mouzinho

**Table S1.** Ordinary least squares regression models of treatment effects on handwashing during the Snack Tests

|  | (1) | (2) | (3) | (4) | (5) | (6) |
| --- | --- | --- | --- | --- | --- | --- |
|  | Wash at  Snack 1 | Wash at  Snack 1 | Wash at  Snack 2 | Wash at  Snack 2 | Wash at  Snack 1&2 | Wash at  Snack 1&2 |
| Treatment: HOPE SOAP**^©^** | 0.02 | 0.04 | 0.11 | 0.06 | 0.10 | 0.10 |
|  | (-0.13 - 0.18) | (-0.12 - 0.20) | (-0.06 - 0.29) | (-0.11 - 0.23) | (-0.08 - 0.27) | (-0.07 - 0.27) |
| Female |  | 0.06 |  | 0.01 |  | 0.10 |
|  |  | (-0.07 - 0.19) |  | (-0.13 - 0.14) |  | (-0.04 - 0.25) |
| Age |  | 0.01 |  | 0.04 |  | 0.01 |
|  |  | (-0.04 - 0.06) |  | (-0.01 - 0.09) |  | (-0.04 - 0.07) |
| Household size |  | -0.03 |  | 0.00 |  | -0.02 |
|  |  | (-0.06 - 0.01) |  | (-0.04 - 0.05) |  | (-0.06 - 0.02) |
| Number of children in hh |  | 0.04 |  | 0.02 |  | 0.06* |
|  |  | (-0.03 - 0.11) |  | (-0.06 - 0.10) |  | (-0.01 - 0.13) |
| Assets (% ownership) |  | 0.45* |  | 0.54** |  | 0.45* |
|  |  | (-0.01 - 0.92) |  | (0.04 - 1.03) |  | (-0.07 - 0.97) |
| Piped water in house |  | 0.25** |  | -0.12 |  | 0.07 |
|  |  | (0.05 - 0.45) |  | (-0.33 - 0.09) |  | (-0.15 - 0.29) |
| HH limits water use: Yes |  | 0.02 |  | 0.12 |  | 0.14 |
|  |  | (-0.14 - 0.17) |  | (-0.05 - 0.29) |  | (-0.04 - 0.31) |
| HH limits water use: No data |  | 0.09 |  | 0.38*** |  | 0.21 |
|  |  | (-0.15 - 0.33) |  | (0.15 - 0.60) |  | (-0.08 - 0.50) |
| Soap observed in HH: Yes |  | 0.00 |  | -0.00 |  | 0.03 |
|  |  | (-0.15 - 0.16) |  | (-0.17 - 0.17) |  | (-0.14 - 0.21) |
| Soap observed in HH: No data |  | 0.12 |  | -0.11 |  | -0.01 |
|  |  | (-0.08 - 0.33) |  | (-0.38 - 0.16) |  | (-0.27 - 0.24) |
| HH received hygiene training: Yes |  | 0.17** |  | 0.24*** |  | 0.19** |
|  |  | (0.02 - 0.33) |  | (0.07 - 0.41) |  | (0.02 - 0.36) |
| HH received hygiene training: No data |  | -0.22 |  | -0.19 |  | -0.14 |
|  |  | (-0.57 - 0.13) |  | (-0.62 - 0.24) |  | (-0.51 - 0.23) |
| Caregiver depressed/anxious: Yes |  | -0.02 |  | 0.13 |  | 0.03 |
|  |  | (-0.17 - 0.13) |  | (-0.05 - 0.30) |  | (-0.14 - 0.21) |
| Difficulty opening tap: Yes |  | -0.03 |  | 0.06 |  | -0.04 |
|  |  | (-0.20 - 0.14) |  | (-0.13 - 0.25) |  | (-0.22 - 0.13) |
| Difficulty opening tap: No data |  | -0.26 |  | -0.39** |  | -0.10 |
|  |  | (-0.67 - 0.15) |  | (-0.69 - -0.09) |  | (-0.40 - 0.19) |
| Hands too small for soap: Yes |  | 0.12 |  | -0.02 |  | 0.06 |
|  |  | (-0.05 - 0.28) |  | (-0.20 - 0.15) |  | (-0.12 - 0.23) |
| Hands too small for soap: No data |  | 0.22 |  | -0.78*** |  | -0.62*** |
|  |  | (-0.17 - 0.61) |  | (-1.01 - -0.55) |  | (-0.86 - -0.38) |
| Can’t reach taps: Yes |  | 0.06 |  | -0.03 |  | -0.00 |
|  |  | (-0.10 - 0.22) |  | (-0.21 - 0.15) |  | (-0.18 - 0.18) |
| Can’t reach taps: No data |  | 0.05 |  | 1.02*** |  | 0.70*** |
|  |  | (-0.36 - 0.47) |  | (0.41 - 1.63) |  | (0.19 - 1.21) |
| Constant | 0.56*** | -0.00 | 0.48*** | -0.14 | 0.39*** | -0.24 |
|  | (0.46 - 0.67) | (-0.46 - 0.46) | (0.36 - 0.60) | (-0.64 - 0.36) | (0.28 - 0.51) | (-0.76 - 0.28) |
| Observations | 230 | 228 | 188 | 187 | 188 | 187 |
| R-squared | 0.00 | 0.12 | 0.01 | 0.17 | 0.01 | 0.13 |
| Notes: 95%CI in parentheses |  |  |  |  |  |  |
| *** p<0.01, ** p<0.05, * p<0.1 |  |  |  |  |  |  |

**Table S2.** Effect of treatment on handwashing after going to the toilet

|  | (1) | (2) | (3) | (4) | (5) |
| --- | --- | --- | --- | --- | --- |
|  | Score 1-10 | Score 1-10 | Score 1-10 | Score 1-10 | Score = 8+ |
|  |  |  |  |  |  |
| Treatment: HOPE SOAP**^©^** | -0.01 | 0.11 | 0.20 | 1.29 | 0.08 |
|  | (-0.69 - 0.67) | (-0.62 - 0.84) | (-0.49 - 0.88) | (-0.33 - 2.91) | (-0.06 - 0.23) |
| Baseline equivalent of dependent variable:  hand washing measure |  |  | 0.22*** | 0.32*** | 0.29*** |
|  |  |  | (0.10 - 0.34) | (0.15 - 0.50) | (0.15 - 0.43) |
| Treatment*Baseline measure |  |  |  | -0.19* |  |
|  |  |  |  | (-0.41 - 0.03) |  |
| Female |  | 0.29 | 0.16 | 0.13 | 0.07 |
|  |  | (-0.27 - 0.84) | (-0.37 - 0.70) | (-0.40 - 0.66) | (-0.05 - 0.18) |
| Age |  | -0.04 | -0.15 | -0.15 | -0.03 |
|  |  | (-0.34 - 0.26) | (-0.43 - 0.14) | (-0.43 - 0.13) | (-0.07 - 0.02) |
| Household size |  | -0.08 | -0.07 | -0.08 | -0.03 |
|  |  | (-0.23 - 0.08) | (-0.22 - 0.08) | (-0.22 - 0.07) | (-0.06 - 0.01) |
| Number of children in hh |  | -0.03 | 0.01 | 0.01 | 0.04 |
|  |  | (-0.34 - 0.27) | (-0.26 - 0.27) | (-0.26 - 0.28) | (-0.02 - 0.11) |
| Assets (% ownership) |  | -1.00 | -1.67 | -1.65 | -0.10 |
|  |  | (-3.21 - 1.22) | (-3.70 - 0.36) | (-3.70 - 0.40) | (-0.56 - 0.35) |
| Piped water in house |  | -0.20 | 0.25 | 0.19 | 0.09 |
|  |  | (-1.26 - 0.86) | (-0.69 - 1.18) | (-0.77 - 1.15) | (-0.11 - 0.30) |
| HH limits water use: Yes |  | 0.14 | 0.22 | 0.18 | 0.06 |
|  |  | (-0.55 - 0.84) | (-0.44 - 0.87) | (-0.48 - 0.84) | (-0.09 - 0.20) |
| HH limits water use: No data |  | -0.23 | -0.05 | -0.12 | 0.05 |
|  |  | (-1.35 - 0.89) | (-1.26 - 1.17) | (-1.27 - 1.02) | (-0.16 - 0.27) |
| Soap observed in HH: Yes |  | 0.26 | 0.24 | 0.17 | -0.00 |
|  |  | (-0.54 - 1.05) | (-0.54 - 1.01) | (-0.59 - 0.93) | (-0.16 - 0.16) |
| Soap observed in HH: No data |  | -0.70 | -0.68 | -0.75 | -0.14 |
|  |  | (-1.91 - 0.50) | (-1.81 - 0.46) | (-1.89 - 0.39) | (-0.35 - 0.07) |
| HH received hygiene training: Yes |  | -0.52 | -0.72* | -0.74** | -0.13 |
|  |  | (-1.34 - 0.30) | (-1.47 - 0.02) | (-1.48 - -0.01) | (-0.28 - 0.03) |
| HH received hygiene training: No data |  | 0.39 | 0.37 | 0.31 | 0.10 |
|  |  | (-1.29 - 2.06) | (-1.29 - 2.03) | (-1.31 - 1.93) | (-0.22 - 0.43) |
| Caregiver depressed/anxious: Yes |  | 0.12 | 0.28 | 0.28 | 0.07 |
|  |  | (-0.56 - 0.80) | (-0.36 - 0.92) | (-0.35 - 0.91) | (-0.07 - 0.21) |
| Difficulty opening tap: Yes |  | -0.49 | -0.69* | -0.74** | -0.16* |
|  |  | (-1.29 - 0.30) | (-1.42 - 0.04) | (-1.47 - -0.02) | (-0.32 - 0.01) |
| Difficulty opening tap: No data |  | -0.53 | 0.38 | 0.21 | 0.22 |
|  |  | (-3.76 - 2.70) | (-2.93 - 3.69) | (-2.72 - 3.13) | (-0.25 - 0.69) |
| Hands too small for soap: Yes |  | -0.32 | -0.09 | -0.10 | -0.02 |
|  |  | (-1.08 - 0.44) | (-0.82 - 0.64) | (-0.84 - 0.64) | (-0.18 - 0.14) |
| Hands too small for soap: No data |  | -2.44 | -1.51 | -1.65 | -0.75*** |
|  |  | (-5.55 - 0.67) | (-4.65 - 1.62) | (-4.41 - 1.11) | (-1.18 - -0.31) |
| Can’t reach taps: Yes |  | -0.07 | -0.10 | -0.12 | -0.01 |
|  |  | (-0.81 - 0.67) | (-0.80 - 0.61) | (-0.83 - 0.58) | (-0.16 - 0.13) |
| Can’t reach taps: No data |  | -0.01 | -0.96 | -1.14 | 0.08 |
|  |  | (-3.20 - 3.19) | (-4.20 - 2.27) | (-3.99 - 1.71) | (-0.38 - 0.54) |
| Constant | 7.42*** | 9.12*** | 8.13*** | 7.74*** | 0.62*** |
|  | (6.90 - 7.95) | (6.50 - 11.73) | (5.63 - 10.64) | (5.12 - 10.36) | (0.20 - 1.04) |
|  |  |  |  |  |  |
| Observations | 249 | 247 | 242 | 242 | 242 |
| R-squared | 0.00 | 0.08 | 0.16 | 0.17 | 0.16 |
| Notes: 95%CI in parentheses |  |  |  |  |  |
| *** p<0.01, ** p<0.05, * p<0.1 |  |  |  |  |  |

**Table S3.** Effect of treatment on handwashing before meals

|  | (1) | (2) | (3) | (4) | (5) |
| --- | --- | --- | --- | --- | --- |
|  | Score 1-10 | Score 1-10 | Score 1-10 | Score 1-10 | Score = 8+ |
|  |  |  |  |  |  |
| Treatment: HOPE SOAP**^©^** | -0.10 | 0.27 | 0.28 | 1.14 | 0.05 |
|  | (-0.86 - 0.65) | (-0.49 - 1.03) | (-0.46 - 1.02) | (-0.46 - 2.73) | (-0.08 - 0.19) |
| Baseline equivalent of dependent variable:  hand washing measure |  |  | 0.13** | 0.22** | 0.10 |
|  |  |  | (0.01 - 0.25) | (0.04 - 0.39) | (-0.05 - 0.26) |
| Treatment*Baseline measure |  |  |  | -0.16 |  |
|  |  |  |  | (-0.40 - 0.09) |  |
| Female |  | -0.18 | -0.19 | -0.22 | -0.05 |
|  |  | (-0.82 - 0.45) | (-0.83 - 0.44) | (-0.86 - 0.41) | (-0.17 - 0.07) |
| Age |  | 0.13 | 0.11 | 0.11 | 0.06** |
|  |  | (-0.17 - 0.43) | (-0.19 - 0.41) | (-0.18 - 0.41) | (0.00 - 0.11) |
| Household size |  | -0.03 | -0.03 | -0.04 | -0.05*** |
|  |  | (-0.19 - 0.13) | (-0.19 - 0.13) | (-0.20 - 0.13) | (-0.08 - -0.01) |
| Number of children in hh |  | -0.01 | 0.04 | 0.04 | 0.07* |
|  |  | (-0.43 - 0.41) | (-0.36 - 0.43) | (-0.36 - 0.45) | (-0.00 - 0.15) |
| Assets (% ownership) |  | -1.48 | -1.60 | -1.67 | -0.09 |
|  |  | (-3.90 - 0.94) | (-4.01 - 0.81) | (-4.04 - 0.70) | (-0.50 - 0.32) |
| Piped water in house |  | 0.60 | 0.59 | 0.62 | 0.14 |
|  |  | (-0.54 - 1.74) | (-0.53 - 1.71) | (-0.49 - 1.74) | (-0.04 - 0.32) |
| HH limits water use: Yes |  | 0.25 | 0.11 | 0.12 | 0.08 |
|  |  | (-0.54 - 1.04) | (-0.68 - 0.89) | (-0.66 - 0.91) | (-0.07 - 0.22) |
| HH limits water use: No data |  | -1.09 | -1.18 | -1.16 | -0.12 |
|  |  | (-2.49 - 0.32) | (-2.64 - 0.28) | (-2.57 - 0.26) | (-0.33 - 0.09) |
| Soap observed in HH: Yes |  | 1.06** | 1.01** | 0.97** | 0.13 |
|  |  | (0.22 - 1.90) | (0.17 - 1.86) | (0.13 - 1.82) | (-0.03 - 0.28) |
| Soap observed in HH: No data |  | 0.77 | 0.85 | 0.80 | 0.12 |
|  |  | (-0.71 - 2.24) | (-0.58 - 2.27) | (-0.63 - 2.24) | (-0.09 - 0.33) |
| HH received hygiene training: Yes |  | 0.16 | 0.18 | 0.18 | -0.03 |
|  |  | (-0.71 - 1.04) | (-0.69 - 1.04) | (-0.68 - 1.04) | (-0.18 - 0.12) |
| HH received hygiene training: No data |  | 0.35 | 0.37 | 0.27 | -0.06 |
|  |  | (-1.23 - 1.93) | (-1.21 - 1.95) | (-1.33 - 1.86) | (-0.39 - 0.26) |
| Caregiver depressed/anxious: Yes |  | -0.43 | -0.34 | -0.38 | -0.07 |
|  |  | (-1.18 - 0.31) | (-1.10 - 0.41) | (-1.13 - 0.37) | (-0.21 - 0.07) |
| Difficulty opening tap: Yes |  | -0.24 | -0.19 | -0.25 | -0.03 |
|  |  | (-1.14 - 0.67) | (-1.10 - 0.71) | (-1.16 - 0.66) | (-0.21 - 0.15) |
| Difficulty opening tap: No data |  | -1.97 | -2.17 | -1.82 | -0.09 |
|  |  | (-6.10 - 2.16) | (-6.28 - 1.95) | (-5.67 - 2.03) | (-0.98 - 0.80) |
| Hands too small for soap: Yes |  | 0.24 | 0.45 | 0.47 | 0.04 |
|  |  | (-0.64 - 1.13) | (-0.44 - 1.34) | (-0.42 - 1.35) | (-0.13 - 0.21) |
| Hands too small for soap: No data |  | -0.52 | 0.20 | 0.07 | -0.18 |
|  |  | (-4.44 - 3.40) | (-3.71 - 4.11) | (-3.51 - 3.65) | (-1.04 - 0.67) |
| Can’t reach taps: Yes |  | -0.33 | -0.39 | -0.45 | -0.10 |
|  |  | (-1.12 - 0.47) | (-1.18 - 0.41) | (-1.24 - 0.34) | (-0.25 - 0.05) |
| Can’t reach taps: No data |  | -0.08 | -0.81 | -0.99 | -0.10 |
|  |  | (-4.12 - 3.96) | (-4.80 - 3.18) | (-4.64 - 2.65) | (-0.99 - 0.79) |
| Constant | 7.01*** | 6.36*** | 5.65*** | 5.32*** | 0.26 |
|  | (6.45 - 7.57) | (3.71 - 9.01) | (2.95 - 8.34) | (2.48 - 8.16) | (-0.18 - 0.70) |
|  |  |  |  |  |  |
| Observations | 249 | 247 | 244 | 244 | 244 |
| R-squared | 0.00 | 0.09 | 0.11 | 0.11 | 0.12 |
| Notes: 95%CI in parentheses |  |  |  |  |  |
| *** p<0.01, ** p<0.05, * p<0.1 |  |  |  |  |  |

**Table S4.** Effect of treatment on general soap usage when washing hands

|  | (1) | (2) | (3) | (4) | (5) |
| --- | --- | --- | --- | --- | --- |
|  | Score 1-10 | Score 1-10 | Score 1-10 | Score 1-10 | Score = 8+ |
|  |  |  |  |  |  |
| Treatment: HOPE SOAP**^©^** | 0.31 | 0.42* | 0.41 | 0.91 | 0.14** |
|  | (-0.27 - 0.89) | (-0.07 - 0.91) | (-0.09 - 0.90) | (-0.89 - 2.70) | (0.03 - 0.24) |
| Baseline equivalent of dependent variable: hand washing measure |  |  | 0.13** | 0.17* | 0.15** |
|  |  |  | (0.01 - 0.26) | (-0.02 - 0.36) | (0.02 - 0.28) |
| Treatment*Baseline measure |  |  |  | -0.07 |  |
|  |  |  |  | (-0.31 - 0.17) |  |
| Female |  | 0.00 | -0.08 | -0.08 | -0.02 |
|  |  | (-0.50 - 0.51) | (-0.59 - 0.42) | (-0.59 - 0.42) | (-0.12 - 0.08) |
| Age |  | -0.05 | -0.09 | -0.09 | -0.01 |
|  |  | (-0.30 - 0.20) | (-0.35 - 0.17) | (-0.34 - 0.16) | (-0.05 - 0.03) |
| Household size |  | -0.05 | -0.07 | -0.06 | -0.03* |
|  |  | (-0.19 - 0.08) | (-0.21 - 0.07) | (-0.21 - 0.08) | (-0.06 - 0.00) |
| Number of children in hh |  | 0.16 | 0.23 | 0.22 | 0.06** |
|  |  | (-0.10 - 0.42) | (-0.05 - 0.51) | (-0.05 - 0.50) | (0.00 - 0.12) |
| Assets (% ownership) |  | -0.29 | -0.45 | -0.43 | -0.09 |
|  |  | (-1.93 - 1.34) | (-2.18 - 1.28) | (-2.14 - 1.29) | (-0.42 - 0.24) |
| Piped water in house |  | 0.21 | 0.02 | 0.06 | 0.10 |
|  |  | (-0.55 - 0.98) | (-0.72 - 0.76) | (-0.68 - 0.81) | (-0.06 - 0.27) |
| HH limits water use: Yes |  | 0.29 | 0.38 | 0.38 | 0.11** |
|  |  | (-0.20 - 0.78) | (-0.13 - 0.89) | (-0.13 - 0.89) | (0.01 - 0.22) |
| HH limits water use: No data |  | -0.94 | -1.11 | -1.11 | -0.16 |
|  |  | (-2.41 - 0.53) | (-2.76 - 0.53) | (-2.77 - 0.55) | (-0.41 - 0.09) |
| Soap observed in HH: Yes |  | 0.09 | 0.08 | 0.06 | -0.10 |
|  |  | (-0.53 - 0.72) | (-0.57 - 0.73) | (-0.60 - 0.73) | (-0.23 - 0.03) |
| Soap observed in HH: No data |  | 0.70* | 0.64 | 0.62 | 0.09 |
|  |  | (-0.10 - 1.51) | (-0.21 - 1.48) | (-0.25 - 1.49) | (-0.07 - 0.25) |
| HH received hygiene training: Yes |  | -0.18 | -0.34 | -0.34 | -0.03 |
|  |  | (-0.80 - 0.44) | (-0.95 - 0.27) | (-0.94 - 0.27) | (-0.15 - 0.08) |
| HH received hygiene training: No data |  | 0.19 | 0.38 | 0.39 | 0.03 |
|  |  | (-1.24 - 1.62) | (-1.08 - 1.83) | (-1.07 - 1.85) | (-0.30 - 0.35) |
| Caregiver depressed/anxious: Yes |  | -0.62** | -0.48 | -0.47 | -0.11* |
|  |  | (-1.20 - -0.04) | (-1.08 - 0.11) | (-1.07 - 0.14) | (-0.23 - 0.00) |
| Difficulty opening tap: Yes |  | -0.32 | -0.33 | -0.38 | 0.01 |
|  |  | (-0.88 - 0.25) | (-0.92 - 0.25) | (-0.97 - 0.22) | (-0.11 - 0.13) |
| Difficulty opening tap: No data |  | -0.05 | -0.48 | -0.36 | 0.20 |
|  |  | (-1.48 - 1.38) | (-2.20 - 1.24) | (-1.97 - 1.25) | (-0.23 - 0.63) |
| Hands too small for soap: Yes |  | -0.11 | -0.05 | -0.06 | -0.08 |
|  |  | (-0.72 - 0.49) | (-0.65 - 0.55) | (-0.66 - 0.55) | (-0.21 - 0.05) |
| Hands too small for soap: No data |  | 0.69 | 1.35 | 1.20 | 0.06 |
|  |  | (-0.70 - 2.09) | (-0.37 - 3.07) | (-0.40 - 2.79) | (-0.34 - 0.47) |
| Can’t reach taps: Yes |  | 0.21 | 0.16 | 0.14 | 0.03 |
|  |  | (-0.36 - 0.78) | (-0.43 - 0.75) | (-0.44 - 0.73) | (-0.08 - 0.15) |
| Can’t reach taps: No data |  | 0.82 | 0.44 | 0.42 | 0.14 |
|  |  | (-0.62 - 2.25) | (-1.26 - 2.14) | (-1.14 - 1.98) | (-0.29 - 0.56) |
| Constant | 8.33*** | 8.77*** | 8.23*** | 7.94*** | 0.79*** |
|  | (7.89 - 8.77) | (6.82 - 10.73) | (5.95 - 10.51) | (5.47 - 10.41) | (0.43 - 1.14) |
|  |  |  |  |  |  |
| Observations | 249 | 247 | 236 | 236 | 236 |
| R-squared | 0.01 | 0.07 | 0.10 | 0.10 | 0.13 |
| Notes: 95%CI in parentheses |  |  |  |  |  |
| *** p<0.01, ** p<0.05, * p<0.1 |  |  |  |  |  |

**Table S5.** Effect of treatment on child health

|  | (1) | (2) | (3) | (4) | (5) | (6) | (7) |
| --- | --- | --- | --- | --- | --- | --- | --- |
|  | Score 1-13 | Score 1-13 | Score 1-13 | Score 1-13 | Any illness | Any illness | Any illness |
|  |  |  |  |  |  |  |  |
| Treatment: HOPE SOAP**^©^** | -0.20 | -0.28 | -0.35 | -0.10 | -0.06 | -0.06 | -0.09 |
|  | (-0.79 - 0.39) | (-0.86 - 0.31) | (-0.96 - 0.26) | (-0.82 - 0.62) | (-0.19 - 0.08) | (-0.18 - 0.07) | (-0.22 - 0.05) |
| Baseline equivalent of dependent variable: hand washing measure |  |  | 0.19** | 0.24** |  |  | 0.16** |
|  |  |  | (0.04 - 0.34) | (0.02 - 0.47) |  |  | (0.01 - 0.31) |
| Treatment*Baseline measure |  |  |  | -0.10 |  |  |  |
|  |  |  |  | (-0.39 - 0.18) |  |  |  |
| Female |  | 0.29 | 0.19 | 0.19 |  | 0.01 | -0.01 |
|  |  | (-0.21 - 0.78) | (-0.33 - 0.70) | (-0.32 - 0.71) |  | (-0.11 - 0.12) | (-0.13 - 0.12) |
| Age |  | -0.11 | -0.19* | -0.21* |  | -0.03 | -0.03 |
|  |  | (-0.31 - 0.09) | (-0.41 - 0.02) | (-0.42 - 0.01) |  | (-0.07 - 0.01) | (-0.07 - 0.02) |
| Household size |  | -0.09 | -0.10 | -0.10 |  | -0.03** | -0.04** |
|  |  | (-0.21 - 0.02) | (-0.23 - 0.03) | (-0.23 - 0.02) |  | (-0.06 - -0.00) | (-0.07 - -0.00) |
| Number of children in hh |  | -0.16 | -0.10 | -0.09 |  | -0.02 | -0.01 |
|  |  | (-0.37 - 0.06) | (-0.31 - 0.11) | (-0.30 - 0.11) |  | (-0.08 - 0.04) | (-0.07 - 0.05) |
| Assets (% ownership) |  | 0.56 | -0.08 | -0.08 |  | 0.35 | 0.32 |
|  |  | (-1.12 - 2.24) | (-1.79 - 1.62) | (-1.79 - 1.62) |  | (-0.08 - 0.77) | (-0.11 - 0.75) |
| Piped water in house |  | -0.78* | -0.75* | -0.74* |  | -0.11 | -0.09 |
|  |  | (-1.61 - 0.05) | (-1.59 - 0.10) | (-1.58 - 0.10) |  | (-0.28 - 0.06) | (-0.26 - 0.08) |
| HH limits water use: Yes |  | 0.60* | 0.76** | 0.75** |  | 0.11 | 0.12 |
|  |  | (-0.01 - 1.22) | (0.13 - 1.38) | (0.13 - 1.38) |  | (-0.03 - 0.25) | (-0.03 - 0.27) |
| HH limits water use: No data |  | -0.28 | 0.12 | 0.07 |  | 0.01 | 0.10 |
|  |  | (-1.09 - 0.52) | (-0.80 - 1.03) | (-0.86 - 1.01) |  | (-0.25 - 0.27) | (-0.14 - 0.33) |
| Soap observed in HH: Yes |  | -0.01 | -0.27 | -0.25 |  | 0.06 | 0.02 |
|  |  | (-0.64 - 0.62) | (-0.93 - 0.39) | (-0.90 - 0.39) |  | (-0.07 - 0.19) | (-0.13 - 0.16) |
| Soap observed in HH: No data |  | 0.01 | -0.11 | -0.12 |  | 0.16 | 0.17 |
|  |  | (-1.06 - 1.08) | (-1.15 - 0.93) | (-1.15 - 0.92) |  | (-0.06 - 0.37) | (-0.04 - 0.38) |
| HH received hygiene training: Yes |  | 0.56** | 0.59** | 0.58** |  | 0.12* | 0.13* |
|  |  | (0.05 - 1.06) | (0.11 - 1.07) | (0.10 - 1.06) |  | (-0.01 - 0.24) | (-0.00 - 0.26) |
| HH received hygiene training: No data |  | 0.83 | 0.62 | 0.83 |  | 0.01 | 0.02 |
|  |  | (-1.25 - 2.92) | (-2.42 - 3.66) | (-2.15 - 3.81) |  | (-0.38 - 0.39) | (-0.41 - 0.45) |
| Caregiver depressed/anxious: Yes |  | 0.48 | 0.54* | 0.55* |  | -0.01 | -0.02 |
|  |  | (-0.10 - 1.05) | (-0.03 - 1.11) | (-0.02 - 1.11) |  | (-0.15 - 0.13) | (-0.16 - 0.12) |
| Difficulty opening tap: Yes |  | -0.05 | -0.12 | -0.11 |  | 0.04 | 0.03 |
|  |  | (-0.67 - 0.57) | (-0.80 - 0.55) | (-0.78 - 0.55) |  | (-0.11 - 0.19) | (-0.12 - 0.18) |
| Difficulty opening tap: No data |  | -3.47** | -1.14* | -1.23** |  | -0.49*** | -0.30** |
|  |  | (-6.46 - -0.47) | (-2.29 - 0.02) | (-2.43 - -0.02) |  | (-0.71 - -0.27) | (-0.57 - -0.02) |
| Hands too small for soap: Yes |  | -0.36 | -0.59* | -0.55* |  | -0.14* | -0.16* |
|  |  | (-1.05 - 0.33) | (-1.22 - 0.05) | (-1.19 - 0.08) |  | (-0.31 - 0.02) | (-0.34 - 0.01) |
| Hands too small for soap: No data |  | -3.55** | -2.07*** | -1.96*** |  | -0.58*** | -0.58*** |
|  |  | (-6.47 - -0.64) | (-2.93 - -1.22) | (-2.92 - -1.00) |  | (-0.77 - -0.39) | (-0.79 - -0.38) |
| Can’t reach taps: Yes |  | -0.07 | -0.07 | -0.06 |  | -0.03 | -0.03 |
|  |  | (-0.65 - 0.51) | (-0.72 - 0.57) | (-0.71 - 0.59) |  | (-0.18 - 0.12) | (-0.19 - 0.13) |
| Can’t reach taps: No data |  | 6.05*** | 1.25 | 1.33 |  | 0.51*** | 0.29 |
|  |  | (3.08 - 9.02) | (-0.94 - 3.44) | (-0.83 - 3.49) |  | (0.28 - 0.74) | (-0.17 - 0.75) |
| Constant | 1.88*** | 2.98*** | 3.18*** | 3.08*** | 0.63*** | 0.82*** | 0.74*** |
|  | (1.43 - 2.33) | (0.90 - 5.07) | (0.93 - 5.43) | (0.89 - 5.28) | (0.53 - 0.72) | (0.42 - 1.21) | (0.31 - 1.17) |
|  |  |  |  |  |  |  |  |
| Observations | 245 | 243 | 209 | 209 | 245 | 243 | 209 |
| R-squared | 0.00 | 0.18 | 0.22 | 0.22 | 0.00 | 0.16 | 0.21 |
| Notes: 95%CI in parentheses |  |  |  |  |  |  |  |
| *** p<0.01, ** p<0.05, * p<0.1 |  |  |  |  |  |  |  |

**Table S6.** Effect of treatment on handwashing and health by correct use of HOPE SOAP**^©^**

|  | (1) | (2) | (3) | (4) | (5) | (6) |
| --- | --- | --- | --- | --- | --- | --- |
|  | Wash hands at both snack tests 0/1 | Wash hands after toilet Score:1-10 | Wash hands before meals Score:1-10 | Uses soap to wash hands Score:1-10 | Illness Score:1-13 | Any illness  0/1 |
|  |  |  |  |  |  |  |
| Intervention (vs Control) |  |  |  |  |  |  |
| HOPE SOAP**^©^** – Not a Toy-Cheat | 0.07 | 0.17 | 0.22 | 0.40 | -0.61* | -0.15** |
|  | (-0.11 - 0.26) | (-0.57 - 0.91) | (-0.60 - 1.05) | (-0.19 - 0.99) | (-1.31 - 0.09) | (-0.31 - -0.00) |
| HOPE SOAP**^©^** –Toy-Cheat | 0.13 | 0.24 | 0.38 | 0.41 | 0.07 | 0.02 |
|  | (-0.09 - 0.35) | (-0.65 - 1.13) | (-0.57 - 1.33) | (-0.25 - 1.06) | (-0.65 - 0.79) | (-0.14 - 0.18) |
| Baseline equivalent of dependent variable: hand washing measure | N/A | 0.22*** | 0.13** | 0.13** | 0.19** | 0.16** |
|  |  | (0.10 - 0.34) | (0.01 - 0.25) | (0.01 - 0.26) | (0.04 - 0.34) | (0.01 - 0.31) |
| Female | 0.10 | 0.16 | -0.20 | -0.08 | 0.19 | -0.01 |
|  | (-0.04 - 0.25) | (-0.37 - 0.70) | (-0.83 - 0.44) | (-0.59 - 0.42) | (-0.32 - 0.70) | (-0.13 - 0.12) |
| Age | 0.01 | -0.15 | 0.11 | -0.09 | -0.21* | -0.03 |
|  | (-0.04 - 0.07) | (-0.43 - 0.13) | (-0.19 - 0.41) | (-0.35 - 0.17) | (-0.42 - 0.01) | (-0.07 - 0.01) |
| Household size | -0.02 | -0.07 | -0.03 | -0.07 | -0.09 | -0.03* |
|  | (-0.06 - 0.02) | (-0.22 - 0.08) | (-0.19 - 0.14) | (-0.21 - 0.07) | (-0.22 - 0.04) | (-0.07 - 0.00) |
| Number of children in hh | 0.06 | 0.01 | 0.03 | 0.23 | -0.11 | -0.01 |
|  | (-0.01 - 0.14) | (-0.26 - 0.27) | (-0.36 - 0.43) | (-0.05 - 0.51) | (-0.32 - 0.11) | (-0.08 - 0.05) |
| Assets (% ownership) | 0.45* | -1.66 | -1.58 | -0.45 | 0.05 | 0.35 |
|  | (-0.07 - 0.97) | (-3.67 - 0.35) | (-3.97 - 0.81) | (-2.16 - 1.27) | (-1.70 - 1.80) | (-0.08 - 0.79) |
| Piped water in house | 0.06 | 0.24 | 0.57 | 0.02 | -0.82* | -0.10 |
|  | (-0.16 - 0.28) | (-0.68 - 1.15) | (-0.55 - 1.69) | (-0.69 - 0.73) | (-1.66 - 0.03) | (-0.27 - 0.07) |
| HH limits water use: Yes | 0.13 | 0.21 | 0.09 | 0.38 | 0.71** | 0.11 |
|  | (-0.04 - 0.31) | (-0.43 - 0.85) | (-0.70 - 0.88) | (-0.14 - 0.89) | (0.10 - 1.31) | (-0.04 - 0.25) |
| HH limits water use: No data | 0.21 | -0.04 | -1.18 | -1.11 | 0.12 | 0.10 |
|  | (-0.07 - 0.50) | (-1.26 - 1.17) | (-2.63 - 0.27) | (-2.76 - 0.53) | (-0.85 - 1.10) | (-0.12 - 0.32) |
| Soap observed in HH: Yes | 0.04 | 0.24 | 1.03** | 0.08 | -0.20 | 0.04 |
|  | (-0.14 - 0.22) | (-0.55 - 1.04) | (0.18 - 1.89) | (-0.57 - 0.74) | (-0.83 - 0.44) | (-0.11 - 0.18) |
| Soap observed in HH: No data | -0.01 | -0.67 | 0.87 | 0.64 | -0.02 | 0.19* |
|  | (-0.27 - 0.25) | (-1.80 - 0.46) | (-0.53 - 2.27) | (-0.21 - 1.49) | (-1.03 - 0.99) | (-0.02 - 0.40) |
| HH received hygiene training: Yes | 0.19** | -0.73* | 0.17 | -0.34 | 0.57** | 0.13* |
|  | (0.02 - 0.35) | (-1.48 - 0.02) | (-0.70 - 1.03) | (-0.96 - 0.28) | (0.09 - 1.06) | (-0.01 - 0.26) |
| HH received hygiene training: No data | -0.15 | 0.37 | 0.37 | 0.38 | 0.68 | 0.04 |
|  | (-0.53 - 0.23) | (-1.28 - 2.02) | (-1.19 - 1.93) | (-1.08 - 1.83) | (-2.28 - 3.65) | (-0.37 - 0.44) |
| Caregiver depressed/anxious: Yes | 0.03 | 0.28 | -0.34 | -0.48 | 0.50* | -0.03 |
|  | (-0.15 - 0.21) | (-0.35 - 0.92) | (-1.09 - 0.40) | (-1.08 - 0.12) | (-0.08 - 1.08) | (-0.17 - 0.11) |
| Difficulty opening tap: Yes | -0.05 | -0.70* | -0.21 | -0.34 | -0.21 | 0.01 |
|  | (-0.23 - 0.13) | (-1.43 - 0.03) | (-1.11 - 0.69) | (-0.93 - 0.26) | (-0.93 - 0.50) | (-0.15 - 0.16) |
| Difficulty opening tap: No data | -0.09 | 0.38 | -2.18 | -0.48 | -0.92 | -0.24* |
|  | (-0.38 - 0.21) | (-2.97 - 3.72) | (-6.38 - 2.01) | (-2.20 - 1.24) | (-2.14 - 0.31) | (-0.53 - 0.04) |
| Hands too small for soap: Yes | 0.05 | -0.09 | 0.45 | -0.05 | -0.60* | -0.17* |
|  | (-0.12 - 0.23) | (-0.82 - 0.64) | (-0.44 - 1.34) | (-0.65 - 0.55) | (-1.22 - 0.03) | (-0.34 - 0.00) |
| Hands too small for soap: No data | -0.59*** | -1.50 | 0.23 | 1.35 | -1.78*** | -0.51*** |
|  | (-0.83 - -0.35) | (-4.65 - 1.65) | (-3.75 - 4.20) | (-0.37 - 3.07) | (-2.80 - -0.76) | (-0.74 - -0.27) |
| Can’t reach taps: Yes | 0.00 | -0.09 | -0.38 | 0.16 | -0.06 | -0.02 |
|  | (-0.17 - 0.18) | (-0.81 - 0.62) | (-1.16 - 0.40) | (-0.43 - 0.74) | (-0.71 - 0.59) | (-0.18 - 0.13) |
| Can’t reach taps: No data | 0.62** | -1.00 | -0.88 | 0.44 | 0.35 | 0.06 |
|  | (0.06 - 1.18) | (-4.28 - 2.29) | (-4.95 - 3.18) | (-1.28 - 2.15) | (-2.21 - 2.91) | (-0.45 - 0.57) |
| Constant | -0.23 | 8.13*** | 5.65*** | 8.23*** | 3.21*** | 0.75*** |
|  | (-0.75 - 0.29) | (5.64 - 10.63) | (2.97 - 8.33) | (5.91 - 10.55) | (0.97 - 5.45) | (0.32 - 1.17) |
|  |  |  |  |  |  |  |
| Observations | 187 | 242 | 244 | 236 | 209 | 209 |
| R-squared | 0.13 | 0.16 | 0.11 | 0.10 | 0.23 | 0.23 |
| Notes: 95%CI in parentheses |  |  |  |  |  |  |
| *** p<0.01, ** p<0.05, * p<0.1 |  |  |  |  |  |  |
